# Food addiction in anorexia nervosa: Implications for the understanding of crossover diagnosis

**DOI:** 10.1002/erv.2897

**Published:** 2022-03-20

**Authors:** Isabel Sanchez, Ignacio Lucas, Lucero Munguía, Lucia Camacho‐Barcia, Mónica Giménez, Jessica Sánchez‐González, Roser Granero, Neus Solé‐Morata, Ashley N. Gearhardt, Carlos Diéguez, Susana Jiménez‐Murcia, Fernando Fernández‐Aranda

**Affiliations:** ^1^ Department of Psychiatry University Hospital of Bellvitge Barcelona Spain; ^2^ CIBER Fisiopatología Obesidad y Nutrición (CIBERObn) Instituto de Salud Carlos III Madrid Spain; ^3^ Psychoneurobiology of Eating and Addictive Behaviors Group Neurosciences Programme Bellvitge Biomedical Research Institute (IDIBELL) Barcelona Spain; ^4^ Department of Psychobiology and Methodology Autonomous University of Barcelona Barcelona Spain; ^5^ Department of Psychology University of Michigan Ann Arbor Michigan USA; ^6^ Department of Physiology CIMUS University of Santiago de Compostela‐Instituto de Investigación Sanitaria Santiago de Compostela Spain; ^7^ Department of Clinical Sciences School of Medicine and Health Sciences University of Barcelona Barcelona Spain

**Keywords:** anorexia nervosa, binge–purge anorexia nervosa, crossover diagnosis, food addiction, restrictive anorexia nervosa

## Abstract

**Objective:**

Food addiction (FA) construct was introduced to reflect abnormal eating patterns that resemble behavioural ones found in substance use disorders. FA has been barely explored in anorexia nervosa (AN). This study evaluated FA occurrence and associated factors in a sample of patients with AN, distinguishing between restrictive and binge–purging subtypes and focussing on the influence of FA in the crossover diagnosis between them.

**Method:**

A sample of 116 patients with AN admitted for treatment seeking at an Bellvitge Hospital Eating Disorders Unit were included (72 restrictive [AN‐R]; 44 binge‐purge AN [AN‐BP]), and eating‐related, personality and psychopathological variables were assessed. Most participants were women (92.2%), mean age 27.1 years old (SD = 10.5).

**Results:**

FA was more prevalent in patients with AN‐BP compared to the AN‐R group (75.0% and 54.2%, respectively). The patients with AN‐R FA+, presented more similar ED symptomatology, general psychopathology and personality traits, with the AN‐BP patients, than with the AN‐R FA‐.

**Conclusions:**

Patients with AN‐R FA+, exhibit more similarities with the AN‐BP subgroup than with the AN‐R FA‐. Thus, it is possible to hypothesise that the presence of FA might be an indicator of the possible crossover from AN‐R to AN‐BP.

## Highlights

- Patients with anorexia nervosa – restrictive subtype (AN‐R) and patients with anorexia nervosa – bulimic‐purgative subtype (AN‐BP) present a prevalence of food addiction (FA) of 54% and 75%, respectively.
- Patients with AN‐R FA+ have more clinical and personality similarities with AN‐BP than with AN‐R FA−.
- The presence of FA could be associated with the possible crossover from AN‐R to AN‐BP.

**Abbreviations**
AN: anorexia nervosa
ANOVA: analysis of variance
AN‐BP: anorexia nervosa – bulimic‐purgative subtype
AN‐R: anorexia nervosa – restrictive subtype
BMI: body mass index
BN: bulimia nervosa
DSM‐5: Diagnostic and Statistical Manual of Mental Disorders—Fifth Edition
ED: eating disorders
EDI‐2: Eating Disorders Inventory‐2
FA: food addiction
FA+: Food Addiction Positive Screening Score
FA−: Food Addiction Negative Screening Score
GSI: Global Severity Index
PSDI: Positive Symptom Distress Index
PST: positive symptom total
SCID‐5: Semi‐Structured Clinical Interview Based on the DSM‐5
SCL‐90R: Symptom Checklist‐Revised
SD: standard deviation
SRAD: substance‐related and addictive disorder
SUD: substance use disorders
TCI‐R: Temperament And Character Inventory‐Revised
YFAS 2.0: Yale Food Addiction Scale 2.0

## 1 INTRODUCTION

Addictive behaviours are persistent and maladaptive behaviours related with impulsive responses and loss of control, and the maintenance of these behaviours despite negative physical, psychological or social repercussions (Grant et al., 2010). According to the fifth edition of the *Diagnostic and Statistical Manual of Mental Disorders* (DSM‐5; American Psychiatric Association, 2013), diagnostic criteria for an addiction‐related disorder include the following: the development of tolerance, abstinence, craving, a high time inversion in obtaining the substance or performing the behaviour, failure in the attempt of stop consuming the substance or carry out the activity despite negative consequences, and the affectation of any other life areas.

Furthermore, the concept of food addiction (FA) has become a focus of growing scientific interest (Fernández‐Aranda et al., 2018). The FA construct was introduced to reflect abnormal eating patterns that resemble behavioural ones found in substance use disorders (SUD; Hauck et al., 2020; Ifland et al., 2009). The common measure of FA is the Yale Food Addiction Scale 2.0 (YFAS 2.0), that applies the substance‐related and addictive disorder (SRAD) criteria from DSM‐5 (American Psychiatric Association, 2013) to the consumption of highly processed foods such as diminished control over consumption, continued use despite negative consequences, withdrawal, tolerance and craving. The presence of FA has been found to overlap with eating disorders (EDs; Fernández‐Aranda et al., 2018).

Initial research of FA construct focussed mostly on addictive eating behaviours in individuals with overweight and obesity (Gearhardt et al., 2014), evaluating FA as a potential explanatory factor for obesity (Ferrario, 2017; Lerma‐Cabrera et al., 2016). As well, several studies have focussed on evaluating the association between FA and the EDs that are more common in people with overweight (de Vries & Meule, 2016; Gearhardt et al., 2012, 2014). High risk for FA has been found in patients with bulimia nervosa (BN; 96% of cases) and binge eating disorder (70%; de Vries & Meule, 2016; Gearhardt et al., 2012; Granero et al., 2018, 2014; Hilker et al., 2016; Meule et al., 2014). Nevertheless, although FA seems to be associated with a propensity to overeat (Guerrero Pérez et al., 2018; Hauck et al., 2017), it does not imply an obesity condition.

There are certain examples within the literature that revealed that FA is not a phenomenon exclusively found in overweight/obese individuals (Granero et al., 2018; Jiménez‐Murcia et al., 2017). Still, there are very few studies of FA in underweight ED patients. A recent German study found that the prevalence of FA was similar in participants who were obese and underweight (17% and 15%, respectively), recruited via the German part of the global panel ‘Lightspeed‐Research’ (Hauck et al., 2020). As well, FA has also been found in individuals with the binge–purge (AN‐BP) and restrictive subtypes (AN‐R) of anorexia nervosa (AN), with rates ranging from 50% for AN‐R to 85.7% for AN‐BP (Granero et al., 2014; Tran et al., 2020). The association of FA with underweight status is unexpected given that this construct aims to measure a phenotype of eating behaviour marked by compulsive, overconsumption of highly processed foods. Given that the hallmark of AN is an under consumption of food to dangerous levels, more research is needed to understand the unexpected endorsement of FA in this ED.

Furthermore, there may be important differences to consider depending on the subtype of AN. The AN‐BP subtype is characterised by a subjective experience of binge eating (e.g., a feeling of loss of control over food intake) despite a small amount of food being consumed (Peat et al., 2009; Rowsell et al., 2016; Wildes et al., 2010). It is possible that individuals with AN‐BP also experience a subjective sense of being addicted to food (although objectively consuming small quantities). In contrast, FA rates may be lower in the AN‐R subtype, where loss of control is not experienced and successful restriction is the main behavioural sign (Brooks et al., 2012; Claes et al., 2010). Prior findings support differences in FA by AN subtype, with consistently higher FA in AN‐BP (86%–88%) versus AN‐R subtypes (50%–62%; Fauconnier et al., 2020; Granero et al., 2014). This difference in FA by AN subtype is also consistent with differences in personality and psychopathology characteristics by subtype. The AN‐BP subtype is usually a more severe psychopathological variant of AN, which is more strongly associated with inhibitory control difficulties, emotion dysregulation, craving and substance use than AN‐R (Fouladi et al., 2015; Mallorquí‐Bagué et al., 2018; Moreno et al., 2009; Rowsell et al., 2016). Thus, AN‐BP appears to be associated with transdiagnostic characteristics also implicated in SRAD disorders, which may be related to the higher endorsement of FA in this subtype. The plausible factors associated with the elevated endorsement of FA in AN‐R are less clear and an important topic of study.

However, it is also important to note that patients with AN are prone to shift from AN‐R to AN‐BP subtypes (and vice versa). Longitudinal studies have found that patients with AN‐R will evolve into AN‐BP in rates ranging from 9.5% to 64% (Eckert et al., 1995; Fichter et al., 2006; Serra et al., 2021; Strober et al., 1997). Even more, crossover form AN‐BP to BN have been found in rates of 54% in 7 years of follow‐up studies (Eddy et al., 2008). These crossovers between diagnoses have been suggested, as well, to be recurrent during the course of the illness (Milos et al., 2005). These studies suggest that ED patients tend to change between different illness states over time (Eckert et al., 1995; Eddy et al., 2008; Fichter et al., 2006; Milos et al., 2005; Serra et al., 2021; Strober et al., 1997); precise information regarding the variables involved in this transition may be helpful to enhance preventive and therapeutic strategies.

The aims of the current study are, first, to evaluate the presence of FA in a sample of patients diagnosed with AN, comparing the prevalence between AN‐R and AN‐BP subtypes; and secondly, to compare the clinical profiles (regarding ED severity, psychopathology and personality) of two groups of AN‐R patients (categorised by the presence or absence of FA) and a group of AN‐BP patients. Having in mind the phenotypic differences between AN‐R and AN‐BP, we hypothesised that it is highly plausible that FA would be more prevalent in AN‐BP than in AN‐R. Furthermore, the association of FA with bulimic‐purgative behaviours could imply that patients with AN‐R FA + would show a more similar clinical profile to the group of AN‐BP patients. The main contribution of the present study is that it could open a new research line to understand the role of FA as a variable involved in the possible crossover from AN‐R to AN‐BP.

## 2 METHODS

### 2.1 Participants

The sample included *n* = 116 adult patients admitted for treatment seeking at the ED Unit at University Hospital of Bellvitge. [Corrections made on 23 March 2022, after first online publication: ‘XXXX’ in the previous sentence has been replaced with ‘University Hospital of Bellvitge’ in this version.] The number of patient who met criteria for AN‐R was *n* = 72, while *n* = 44 had AN‐BP. Age ranged from 18 to 66 years‐old (mean = 27.1, SD = 10.4). Most participants were women (92.2%), single (85.3%), with secondary education levels (43.1%), unemployed or students (56.9%) and within mean‐low or low social position indexes levels (72.4%). Mean age of onset of the AN was 17.8 years‐old (SD = 4.7) and mean duration of the AN was 9.2 years (SD = 10.2). Mean for BMI upon arrival to the treatment unit was 16.7 kg/m^2^ (SD = 1.5). Table S1 (supplementary material) shows the description stratified by the AN subtype (AN‐R vs. AN‐BP). No differences between groups emerged for the sociodemographic features, age of onset and duration of the disorder. BMI was higher in the AN‐BP patients compared to AN‐R (17.1 vs. 16.4 kg/m^2^; *p* = 0.007).

### 2.2 Procedure

Recruitment date was between May 2016 and June 2019. The inclusion criteria were age (18 years or older) and diagnosis of AN. The diagnosis of ED was made by an expert clinical psychologist, through a face‐to‐face semi‐structured clinical interview based on the DSM‐5 criteria for ED (SCID‐5; First et al., 2015). The Ethical Commitee Board of the University Hospital of Bellvitge approved this study, and all the participants signed a written informed consent before participation.

### 2.3 Measures

#### 2.3.1 Eating Disorders Inventory‐2 (EDI‐2), *Spanish version* (Garner, 1998)

This is a 91‐item self‐rating questionnaire that evaluates different attitudinal and behavioural dimensions related to the psychopathological state and personality traits. It consists of 11 subscales (dimensions): drive for thinness, body dissatisfaction, bulimia, ineffectiveness, perfectionism, interpersonal distrust, interoceptive awareness, maturity fears, asceticism, impulse regulation and social insecurity. For this study, the internal consistency of the EDI‐2 subscales ranged from *α* = 0.706 to *α* = 0.898, and for the total score was *α* = 0.956.

#### 2.3.2 Yale Food Addiction Scale 2.0 (Gearhardt et al., 2016)

This is a self‐report scale that assesses addictive‐like eating behaviour through different addictive disorders criteria (SRAD) DSM‐5 (American Psychiatric Association, 2013). It consists of 35 items on an 8‐points Likert scale (0 = never, 7 = every day). The Spanish validation of the Yale Food Addiction Scale (YFAS) 2.0 version was used in the current sample (Granero et al., 2018) The instrument allows to obtain a dimensional total score reflecting the number of total diagnostic criteria presented for each participant (from 0 to 11), with three severity cut‐offs: mild (2–3 symptoms), moderate (4–5 symptoms) and severe (6–11 symptoms); and an FA classification (present or absent) attending both the number of symptoms presented (a minimum of 2) and self‐reported measures related to clinical impairment and distress (i.e., obesity, harm avoidance, etc.). For this study, the internal consistency of the YFAS 2.0 total score was *α* = 0.959.

#### 2.3.3 Symptom Checklist‐Revised (SCL‐90R; Derogatis, 1994)

This is a self‐reported 90‐item instrument designed to assess global symptoms of psychopathology and distress through different symptom dimensions (9): somatisation, obsessive‐compulsive, interpersonal sensitivity, depression, anxiety, hostility, phobic anxiety, paranoid ideation, and psychoticism. The test provides three global measures: Global Severity Index (GSI), Positive Symptom Total (PST), and Positive Symptom Distress Index (PSDI). For this study the GSI variable was evaluated. We used the validation in Spanish population (Derogatis, 2002). For this study, the internal consistency of the SCL‐90‐R subscales ranged from *α* = 0.816 to *α* = 0.918, for the GSI was *α* = 0.979, for the PST was *α* = 0.979, and for the PSDI was *α* = 0.979.

#### 2.3.4 Temperament and Character Inventory‐Revised (Cloninger, 1999)

It is a questionnaire that evaluates different temperamental and character dimensions (novelty seeking, harm avoidance, reward dependence, persistence, self‐directedness, cooperativeness, and self‐transcendence) in order to establish a personality profile. In this study, the Spanish version of Temperament and Character Inventory‐Revised (TCI‐R; Gutiérrez‐Zotes et al., 2004) was used which has demonstrated adequate psychometric properties. For this study, the internal consistency of the TCI‐R subscales ranged from *α* = 0.832 to *α* = 0.908.

### 2.4 Statistical analysis

Statistical analysis was carried out with Stata17 for Windows (Stata Press, 2019). The comparison between the groups of the study was based on chi‐square tests (χ^2^) for categorical variables and analysis of variance for quantitative measures (ANOVA, defining the Fisher's Least Difference test for multiple‐pairwise comparisons). For these analyses, the effect size for proportion differences and mean differences was measured through the standardised Cohen's‐*h* and Cohen's‐*d* coefficients (low‐poor effect size was considered for |*values*|>0.2, mild‐moderate for |*values*|>0.5 and large‐high for |*values*|>0.8; Kelley & Preacher, 2012). In addition, the potential increase in the Type‐I error due to the multiple statistical comparisons was controlled with Finner procedure, a familywise error rate stepwise method which has demonstrated good reliability and more powerful capacity than classical Bonferroni correction (Finner & Roters, 2001).

## 3 RESULTS

### 3.1 Food addiction prevalence and symptom count

Table 1 includes the distribution of the FA measures for the total sample and the comparison of the two groups defined for the AN subtype (AN‐R vs. AN‐BP). The proportion of patient who met criteria for each SRAD criterion was higher among AN‐BP patients compared to AN‐R. The criterion with the highest likelihood to be met was reporting ‘clinically significant impairment or distress’ (with a prevalence of 81.8% within AN‐BP and 63.9% within AN‐R). The criteria with the lowest prevalence for AN‐BP patients were ‘failure to fulfil major rule obligations’ and ‘use in physically hazardous situations’ (40.9%), while AN‐R patients reported as the lowest prevalence ‘use in physically hazardous situations’ (11.1%). The prevalence of FA positive screening score was 75.0% for AN‐BP compared to 54.2% for AN‐R (*p* < 0.001), and the mean for the total number of SRAD criteria was 6.0 for AN‐BP compared to 3.0 for AN‐R (*p* < 0.001).

**TABLE 1:** FA prevalence and symptom count in the total sample and by AN subtypes

	Total (*n* = 116*)*		AN‐R (*n* = 72)		AN‐BP (*n* = 44)			
FA: SRAD criteria	*N*	%	*N*	%	*n*	%	*p*	*|d|*
Substance taken in larger amount	45	38.8%	19	26.4%	26	59.1%	**<0.001** ^a^	**0.67** ^b^
Persistent desire	35	30.4%	14	19.4%	21	48.8%	**0.001** ^a^	**0.63** ^b^
Much time‐activity to obtain, use, recover	55	47.4%	26	36.1%	29	65.9%	**0.002** ^a^	**0.61** ^b^
Social or occupational affectation	71	61.2%	38	52.8%	33	75.0%	**0.017** ^a^	**0.50** ^b^
Use continues despite consequences	57	49.6%	27	38.0%	30	68.2%	**0.002** ^a^	**0.61** ^b^
Tolerance	36	31.0%	16	22.2%	20	45.5%	**0.009** ^a^	**0.50** ^b^
Withdrawal symptoms	64	55.2%	34	47.2%	30	68.2%	**0.028** ^a^	0.43^b^
Continued use despite social problems	30	25.9%	11	15.3%	19	43.2%	**0.001** ^a^	**0.63** ^b^
Failure to fulfil major rule obligations	27	23.3%	9	12.5%	18	40.9%	**<0.001** ^a^	**0.67** ^b^
Use in physically hazardous situations	26	22.4%	8	11.1%	18	40.9%	**<0.001** ^a^	**0.71** ^b^
Craving, or a strong desire or urge to use	33	28.4%	13	18.1%	20	45.5%	**0.002** ^a^	**0.60** ^b^
Clinically significant impairment‐distress	82	70.7%	46	63.9%	36	81.8%	**0.035** ^a^	0.41
FA: Screening group	*N*	%	*N*	%	*n*	%	*P*	*|d|*
Positive score	72	62.1%	39	54.2%	33	75.0%	**0.025** ^a^	0.44
FA: Severity group	*N*	*%*	*N*	*%*	*n*	*%*	*P*	*|d|*
Null (negative screening)	44	37.9%	33	45.8%	11	25.0%	**<0.001** ^a^	0.44
Mild	24	20.7%	19	26.4%	5	11.4%		0.39
Moderate	18	15.5%	11	15.3%	7	15.9%		0.02
Severe	30	25.9%	9	12.5%	21	47.7%		**0.80** ^b^
FA dimensional measure	Mean	SD	Mean	SD	Mean	SD	*p*	*|d|*
YFAS total score	4.13	3.34	2.99	2.51	6.00	3.69	**<0.001** ^a^	**0.95** ^b^

*Note*: For the chi‐square tests, all the cells have expected count equal or higher than 5.

Abbreviations: AN‐BP, anorexia–bulimic/purgative subtype; AN‐R, anorexia–restrictive subtype; FA, food addiction; SD, standard deviation; SRAD, substance‐related and addictive disorders; YFAS, Yale Food Addiction Scale.

^a^ Bold: significant comparison (*p* < 0.05) *p*‐values include Finner correction for multiple statistical tests.

^b^ Bold: effect size into the moderate‐mild (*|d|*> 0.50) to large‐high (*|d| *> 0.80) range.

### 3.2 Clinical and personality variables comparison between the groups

**FIGURE 1:**
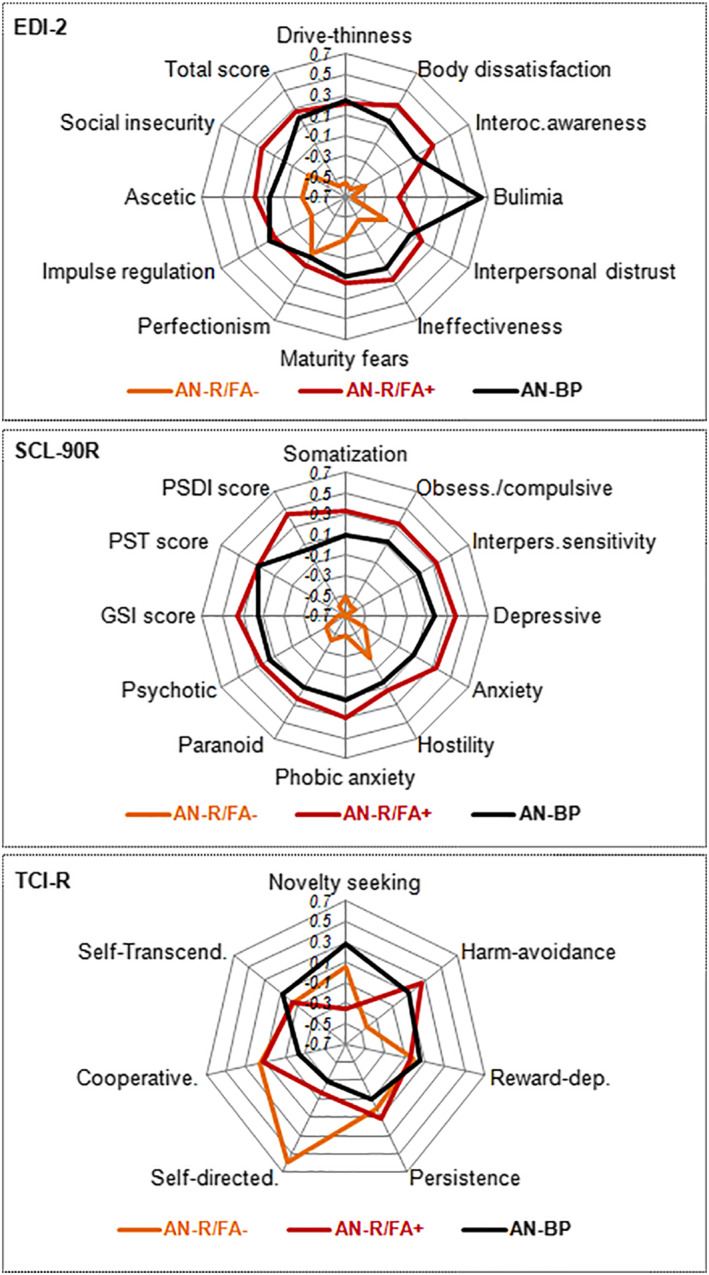
Radar‐charts comparing the clinical profiles between the groups. *Z*‐standardised means are plotted. Sample size: *n* = 112. AN‐R, anorexia – restrictive subtype; AN‐BP, anorexia – bulimic purgative subtype; FA, food addiction; EDI‐2, Eating Disorders Inventory‐2; SCL‐90‐R, Symptom Checklist‐Revised; GSI, Global Severity Index; PSDI, Positive Symptom Distress Index; PST, positive symptom total; TCI‐R, Temperament and Character Inventory‐Revised. *Bold: significant comparison (0.05). †Bold: effect size into the moderate‐mild (|*d*| > 0.50) to large‐high (|*d*| > 0.80) range

Table 2 displays the results of the ANOVA procedures comparing the clinical profiles (EDI‐2, SCL‐90R and TCI‐R mean scores) between the three groups of the study (see also the radar charts in Figure 1). These results indicate that AN‐R with FA is similar to AN‐BP, and differences only were found for the bulimia symptom level and the novelty seeking level (higher means among the AN‐BP patients). However, compared to these two conditions (AN‐R FA+ and AN‐BP), the patients within the AN‐R without FA reported lower ED symptom levels (lower means in several EDI‐2 scales), better psychopathology state (lower means in the SCL‐90R, except for hostility), lower level in the harm avoidance temperament dimension and higher level in the self‐directedness character dimension.

**TABLE 2:** Clinical and personality variables comparison between the groups

	AN‐R FA− (*n* = 33)		AN‐R FA+ (*n* = 39)		AN‐BP (*n* = 44)		AN‐R FA− versus AN‐R FA+		AN‐R FA− versus AN‐BP		AN‐R FA+ versus AN‐BP	
	Mean	SD	Mean	SD	Mean	SD	*p*	*|d|*	*P*	*|d|*	*p*	*|d|*
EDI‐2: Drive for thinness	6.39	6.73	11.90	6.19	12.09	7.05	**0.001** ^a^	**0.85** ^b^	**<0.001** ^a^	**0.83** ^b^	0.895	0.03
EDI‐2: Body dissatisfaction	7.52	6.68	14.13	6.77	12.86	6.13	**<0.001** ^a^	**0.98** ^b^	**0.001** ^a^	**0.83** ^b^	0.379	0.20
EDI‐2: Interoceptive awareness	6.91	7.47	12.69	7.61	11.18	6.60	**0.001** ^a^	**0.77** ^b^	**0.011** ^a^	**0.61** ^b^	0.342	0.21
EDI‐2: Bulimia	0.82	1.40	2.74	2.77	6.18	5.17	**0.028** ^a^	**0.88** ^b^	**<0.001** ^a^	**1.42** ^b^	**<0.001** ^a^	**0.83** ^b^
EDI‐2: Interpersonal distrust	5.27	5.35	7.38	5.27	6.68	5.03	0.089	0.40	0.242	0.27	0.540	0.14
EDI‐2: Ineffectiveness	7.24	7.48	12.23	7.46	11.32	6.94	**0.004** ^a^	**0.67** ^b^	**0.016** ^a^	**0.57** ^b^	0.569	0.13
EDI‐2: Maturity fears	6.30	4.93	9.13	7.46	8.70	6.23	0.063	0.45	0.104	0.43	0.762	0.06
EDI‐2: Perfectionism	5.97	4.86	6.51	4.22	6.09	5.04	0.628	0.12	0.912	0.02	0.686	0.09
EDI‐2: Impulse regulation	3.67	4.73	5.97	5.32	6.32	5.79	0.071	0.46	**0.033** ^a^	**0.50** ^b^	0.771	0.06
EDI‐2: Ascetic	5.06	5.34	7.00	3.93	6.41	3.61	0.057	0.41	0.173	0.30	0.530	0.16
EDI‐2: Social insecurity	6.45	5.37	9.21	5.46	7.91	4.79	**0.027** ^a^	**0.51** ^b^	0.226	0.29	0.259	0.25
EDI‐2: Total score	61.61	43.23	98.90	41.92	95.75	39.37	**<0.001** ^a^	**0.88** ^b^	**<0.001** ^a^	**0.83** ^b^	0.730	0.08
SCL‐90R: Somatisation	1.14	0.89	1.96	0.98	1.72	0.86	**<0.001** ^a^	**0.87** ^b^	**0.007** ^a^	**0.66** ^b^	0.251	0.25
SCL‐90R: Obsessive/compulsive	1.14	0.92	1.98	0.74	1.81	0.84	**<0.001** ^a^	**1.01** ^b^	**0.001** ^a^	**0.76** ^b^	0.346	0.22
SCL‐90R: Interpersonal sensitivity	1.24	1.08	2.15	0.92	1.95	0.83	**<0.001** ^a^	**0.91** ^b^	**0.001** ^a^	**0.74** ^b^	0.341	0.22
SCL‐90R: Depressive	1.48	1.09	2.53	0.74	2.33	0.83	**<0.001** ^a^	**1.13** ^b^	**<0.001** ^a^	**0.88** ^b^	0.301	0.26
SCL‐90R: Anxiety	1.18	1.07	1.98	0.92	1.73	0.94	**0.001** ^a^	**0.80** ^b^	**0.016** ^a^	**0.54** ^b^	0.232	0.28
SCL‐90R: Hostility	1.01	1.16	1.36	0.86	1.27	0.94	0.127	0.35	0.247	0.25	0.664	0.10
SCL‐90R: Phobic anxiety	0.43	0.52	1.17	1.10	1.01	0.88	**0.001** ^a^	**0.86** ^b^	**0.005** ^a^	**0.80** ^b^	0.406	0.16
SCL‐90R: Paranoid	0.93	0.93	1.57	0.97	1.45	0.88	**0.005** ^a^	**0.67** ^b^	**0.018** ^a^	**0.57** ^b^	0.552	0.13
SCL‐90R: Psychotic	0.82	0.76	1.40	0.82	1.33	0.72	**0.002** ^a^	**0.74** ^b^	**0.004** ^a^	**0.69** ^b^	0.666	0.09
SCL‐90R: GSI score	1.11	0.87	1.90	0.68	1.73	0.70	**<0.001** ^a^	**1.00** ^b^	**<0.001** ^a^	**0.79** ^b^	0.311	0.24
SCL‐90R: PST score	44.76	25.17	64.46	12.62	64.32	14.99	**<0.001** ^a^	**0.99** ^b^	**<0.001** ^a^	**0.94** ^b^	0.971	0.01
SCL‐90R: PSDI score	1.97	0.61	2.60	0.57	2.36	0.51	**<0.001** ^a^	**1.07** ^b^	**0.003** ^a^	**0.69** ^a^	0.051	0.45
TCI‐R: Novelty seeking	95.52	14.12	88.03	17.97	99.59	19.92	0.078	0.46	0.322	0.24	**0.004** ^a^	**0.61** ^b^
TCI‐R: Harm avoidance	106.52	21.02	120.85	20.01	117.57	19.80	**0.003** ^a^	**0.70** ^b^	**0.019** ^a^	**0.54** ^b^	0.463	0.16
TCI‐R: Reward dependence	96.48	15.72	96.00	16.26	97.52	16.83	0.900	0.03	0.783	0.06	0.672	0.09
TCI‐R: Persistence	117.70	21.26	119.90	18.28	115.36	23.98	0.665	0.11	0.637	0.10	0.338	0.21
TCI‐R: Self‐directedness	138.42	19.79	121.77	20.80	118.84	21.19	**0.001** ^a^	**0.82** ^b^	**<0.001** ^a^	**0.96** ^b^	0.521	0.14
TCI‐R: Cooperativeness	137.67	16.46	137.13	14.80	131.66	14.98	0.882	0.03	0.092	0.38	0.108	0.37
TCI‐R: Self‐transcendence	60.97	13.23	61.23	13.32	62.86	16.01	0.939	0.02	0.568	0.13	0.607	0.11

Abbreviations: AN‐R, anorexia – restrictive subtype; AN‐BP, anorexia – bulimic purgative subtype; FA, food addiction; EDI‐2, Eating Disorders Inventory‐2; GSI, Global Severity Index; SCL‐90‐R: Symptom Checklist‐Revised; SD, standard deviation; PSDI, Positive Symptom Distress Index; PST, positive symptom total; TCI‐R, Temperament and character inventory‐revised.

^a^ Bold: significant comparison (*p* < 0.05) *p*‐values include Finner correction for multiple statistical tests.

^b^ Bold: effect size into the moderate‐mild (*|d| *> 0.50) to large‐high (*|d| *> 0.80) range.

## 4 DISCUSSION

The aims of this research were, in the first place, to assess FA occurrence in different subtypes of AN, and in the second place, to study clinical and personality variables between two different profiles of AN‐R, classified by the presence of FA, and AN‐BP. First, it is important to note that the prevalence of FA in AN patients was higher than the common prevalence of FA found in previous studies with healthy control population (Meule & Gearhardt, 2019). This is consistent with the idea that FA is not exclusive to overweight population (Granero et al., 2018; Jiménez‐Murcia et al., 2017), but a transdiagnostic problem that affects people with high concerns about food who could have binge episodes or even could end up restricting its intake (Brooks et al., 2012; Claes et al., 2010; Granero et al., 2014; Hauck et al., 2020; Tran et al., 2020). Moreover, AN‐BP patients presented higher prevalence of FA than the AN‐R subtype; being all the FA criteria higher in the AN‐BP sample than in the AN‐R one. As expected, the presence of addictive behaviours towards food was more common in those AN patients who also showed bulimic symptomatology (Fauconnier et al., 2020; Granero et al., 2014). Furthermore, bulimic symptomatology had also been related with comorbidities with substance use disorders and behavioural addictions (Becker & Grilo, 2015; Jiménez‐Murcia et al., 2013; Keski‐Rahkonen, 2021; Munn‐Chernoff & Baker, 2016).

In relation to the second hypothesis, the AN‐R FA+ group showed very similar scores to the AN‐BP group in their ED symptomatology, general psychopathology and personality traits. These similarities were not present between the AN‐R FA− group and AN‐BP patients. The commonalities in the clinical profile of patients with AN‐R FA+ and patients with AN‐BP may suggest that FA could be a variable associated with the possible crossover from AN‐R to AN‐BP. Previous studies showed that a significant proportion of patients diagnosed with AN‐R eventually change to AN‐BP (Eckert et al., 1995; Fichter et al., 2006; Serra et al., 2021; Strober et al., 1997). Also, AN‐R FA+ and AN‐BP groups showed similar personality profiles, with differences from AN‐R FA− in self‐directedness and harm avoidance. Both personality traits had been associated with a higher risk of having an addictive disorder (Granero et al., 2018; Jiménez‐Murcia et al., 2017; Wolz et al., 2016). The only personality trait that showed significant differences between the AN‐R FA+ group and AN‐BP patients is novelty seeking. Addictive behaviours are often associated to high scores in novelty seeking, nevertheless, patients with AN‐R usually present significant low scores in this dimension (Atiye et al., 2015). Overall, having information about variables that are associated to bulimic‐purgative symptomatology in AN would be essential to adapt the treatment of the disorder. Having this in mind, the FA assessment of AN patients could help to prevent the onset of these behaviours and the transition AN‐BP.

### 4.1 Limitations and future research lines

All the conclusions derived from this study must be interpreted taking in account some limitations. First, this is a transversal study that compared the clinical profiles of AN patients. So, all the hypothesis about the possible influence of FA in developing a more severe profile of AN should be confirmed by future studies using longitudinal methodology. Second, the low sample sizes when comparing between the groups could limit the generalisation of the results. Third, this is one of the few studies to date that evaluated the presence of FA in a population diagnosed with AN, further validation work would be necessary to define precisely the FA symptomatology measured with the YFAS in a population characterised by food restriction. Additionally, further research of the association between AN and FA is needed to explain their co‐occurrence.

## 5 CONCLUSIONS

Overall, the prevalence of FA was higher in patients with AN‐BP than in AN‐R ones. This finding is congruent with the more severe eating symptomatology present in AN‐BP, as the perception of loss of control over food consumption or the occurrence of bulimic episodes. Besides, the main finding of this study was the similar clinical profiles of AN‐R patients with FA and AN‐BP patients, regarding their ED symptomatology, general psychopathology and personality traits. The phenotypical features of AN‐R patients who also present FA, seem to be more similar to AN‐BP than to the AN‐R without FA.

## CONFLICT OF INTEREST

Fernando Fernández‐Aranda received consultancy honoraria from Novo Nordisk and editorial honoraria as EIC from Wiley. The rest of the authors declare no conflict of interest. The funders had no role in the design of the study; in the collection, analyses or interpretation of data; in the writing of the manuscript, or in the decision to publish the results.

## PATIENT CONSENT STATEMENT

Informed consent was obtained from all individual participants included in the study.

## AUTHOR CONTRIBUTIONS

Isabel Sanchez, Jessica Sánchez‐González, Susana Jiménez‐Murcia and Fernando Fernández‐Aranda contributed to the development of the study concept and design. Roser Granero performed the statistical analysis. Lucia Camacho‐Barcia and Jessica Sánchez‐González aided with data collection. Isabel Sanchez, Ignacio Lucas, Lucero Munguía and Mónica Giménez aided with interpretation of data and the writing of the manuscript. Ashley Gearhard, Carlos Diéguez, Susana Jiménez‐Murcia and Fernando Fernández‐Aranda aided with supervision, review and editing of the manuscript.

## Supporting information

Supporting Information S1

## Data Availability

The datasets generated during and/or analysed during the current study are not publicly available due to ethical restrictions in order to protect the confidentiality of the participants, but are available from the corresponding author on reasonable request.
