# Supplementary material for: Food addiction in anorexia nervosa: Implications for the understanding of crossover diagnosis
Source: Eur Eat Disord Rev. 2022 Mar 20;30(3):278–88. doi: 10.1002/erv.2897 (PMC9314799; doi:10.1002/erv.2897)
Supplement: Supplementary file 1 — Supporting Information S1 [file ERV-30-278-s001.docx]

***Table S1 (supplementary material)*** *Descriptive of the sample*

|  | Total  (*n=116)* | | AN-R  (*n=72)* | | AN-BP  (*n=44)* | |  |
| --- | --- | --- | --- | --- | --- | --- | --- |
|  | *n* | *%* | *n* | *%* | *n* | *%* | *p* |
| Sex Women | 107 | 92.2% | 68 | 94.4% | 39 | 88.6% | .257 |
| Men | 9 | 7.8% | 4 | 5.6% | 5 | 11.4% |  |
| Civil status Single | 99 | 85.3% | 62 | 86.1% | 37 | 84.1% | .652 |
| Married/Partner | 16 | 13.8% | 9 | 12.5% | 7 | 15.9% |  |
| Divorced/Separated | 1 | 0.9% | 1 | 1.4% | 0 | 0.0% |  |
| Education Primary or less | 42 | 36.2% | 24 | 33.3% | 18 | 40.9% | .683 |
| Secondary | 50 | 43.1% | 33 | 45.8% | 17 | 38.6% |  |
| University | 24 | 20.7% | 15 | 20.8% | 9 | 20.5% |  |
| Employment Unemployed-student | 66 | 56.9% | 43 | 59.7% | 23 | 52.3% | .432 |
| Employed | 50 | 43.1% | 29 | 40.3% | 21 | 47.7% |  |
| Social index High | 1 | 0.9% | 0 | 0.0% | 1 | 2.3% | .380 |
| Mean-high | 16 | 13.8% | 12 | 16.7% | 4 | 9.1% |  |
| Mean | 15 | 12.9% | 8 | 11.1% | 7 | 15.9% |  |
| Mean-low | 45 | 38.8% | 30 | 41.7% | 15 | 34.1% |  |
| Low | 39 | 33.6% | 22 | 30.6% | 17 | 38.6% |  |
|  | *Mean* | *SD* | *Mean* | *SD* | *Mean* | *SD* | *p* |
| Chronological age (years-old) | 27.06 | 10.45 | 26.36 | 10.50 | 28.20 | 10.40 | .359 |
| Age of onset of AN (years-old) | 17.84 | 4.70 | 18.38 | 4.87 | 16.98 | 4.33 | .121 |
| Duration of AN (years) | 9.23 | 10.19 | 8.01 | 10.17 | 11.24 | 10.03 | .098 |
| BMI (current, kg/m^2^) | 16.66 | 1.49 | 16.37 | 1.65 | 17.13 | 1.03 | **.007*** |

*Note.* AN-R: anorexia – restrictive subtype. AN-BP: anorexia – bulimic/purgative subtype.

SD: standard deviation. *Bold: significant comparison (.05).
